# A Review on Plants Used for Improvement of Sexual Performance and Virility

**DOI:** 10.1155/2014/868062

**Published:** 2014-08-18

**Authors:** Nagendra Singh Chauhan, Vikas Sharma, V. K. Dixit, Mayank Thakur

**Affiliations:** ^1^Department of Pharmaceutical Sciences, Dr. Harisingh Gour Central University, Sagar, Madhya Pradesh 470003, India; ^2^Drugs Testing Laboratory Avam Anusandhan Kendra, GE Road, Raipur, Chhattisgarh 492010, India; ^3^Institute for Laboratory Medicine Clinical Chemistry, and Pathobiochemistry, Charite Universitätsmedizin, Campus Virchow Klinikum, Augustenburger Platz 1, 12200 Berlin, Germany

## Abstract

The use of plant or plant-based products to stimulate sexual desire and to enhance performance and enjoyment is almost as old as the human race itself. The present paper reviews the active, natural principles, and crude extracts of plants, which have been useful in sexual disorders, have potential for improving sexual behaviour and performance, and are helpful in spermatogenesis and reproduction. Review of refereed journals and scientific literature available in electronic databases and traditional literature available in India was extensively performed. The work reviews correlation of the evidence with traditional claims, elucidation, and evaluation of a plausible concept governing the usage of plants as aphrodisiac in total. Phytoconstituents with known structures have been classified in appropriate chemical groups and the active crude extracts have been tabulated. Data on their pharmacological activity, mechanism of action, and toxicity are reported. The present review provides an overview of the herbs and their active molecule with claims for improvement of sexual behaviour. A number of herbal drugs have been validated for their effect on sexual behavior and fertility and can therefore serve as basis for the identification of new chemical leads useful in sexual and erectile dysfunction.

## 1. Introduction

Male reproductive capacity was found to be deficient in nearly 50% of infertile couples according to a study carried out by the World Health Organization in 1987. Although further figures for this decade are still awaited, it is certain that stressful life style has enhanced the number of subject's suffering from one form of sexual dysfunction or the other. Main factors that decrease the probability of conception in the female partner are frequently congenital, immunological, iatrogenic, or endocrine cause. Oligozoospermia, sexual, and ejaculatory dysfunction are further responsible for inability to conceive in numerous cases [1]. Although many synthetic drugs are available and/or used to treat these problems, some of the drawbacks for these drugs include them being expensive and also their ability to provoke serious adverse effects, effective natural treatments are therefore still in demand. Even if many of the plants or natural products claim to prove their effectiveness without scientific evidence, a number of them are active and possess biological activity, proven by scientific data. Moreover, there is a dearth of systematic review of scientific literature on experimental evidence generated for medicinal plants useful in treating erectile dysfunction and there is a need for in depth pharmacological evaluation [2].

Advancement in the understanding of pharmacological basis of erectile and sexual functions at molecular levels is turning out to be stepping stones towards isolating the crucial physiologic factors involved in sexual arousal, thus helping to narrow down the search for aphrodisiac substances of choice. Many people do not believe in love potions or aphrodisiacs, but countless numbers of men and women have used them down through the centuries, and there is clear proof that they are still in use today. The skepticism towards the concept of aphrodisiac is not unjustified, although a systematic evaluation and compilation of scientific information may provide a basis for the evidence-based utilization of herbal drugs for treatment of sexual dysfunction in general. The present review is an attempt to consummate the available scientific information on various herbal drugs, which have been evaluated for their effect on sexual performance and functionality. The review also includes known evidences collected for the involvement of herbal drugs on neural, nitric oxide and hormone-dependent mechanisms and their role on sexual functions. A number of plants have been discussed in detail and a few others are only tabulated; a major criterion for this arrangement was the ethnopharmacological relevance of the plant in the Ayurvedic system of medicine. Nonetheless, it is very important to mention that this does not entail a grading system for the plants described in the paper and some of the plants only listed in tabular form may also be of high scientific relevance.

## 2. Historical Background

The word “Aphrodisiac” is derived from “Aphrodite” the Greek goddess of love. By definition aphrodisiacs are the substance, which stimulate sexual desire (Greek-Aphrodisiakos-sexual) [3]. A variety of plants have been used as sex stimulants or sexual performance enhancer in traditional systems of medicine of various countries [4–6]. Practitioners of Ayurveda the traditional system of medicine in India recognized the vital importance of virility and formulated Vajikarna therapy [7] (Table 1). Modern day concept for the term “aphrodisiac” can be considered close to the Vajikarna concept defined in traditional texts of Ayurvedic medicine.

## 3. Vajikaran in Ayurvedic Texts

**Figure 1:**
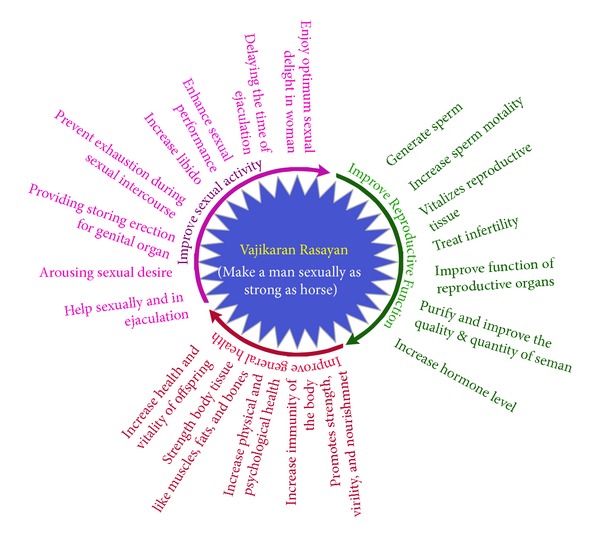
Action of Vajikaran Rasayana.

Vajikaran as a concept has been defined in the* Rig Veda* and the* Yajurveda*, the first written texts of medicine, in Ayurveda. Vajikarana herbs are also the basis for therapies recommended in* Kamasutra*, a treatise defining methods for appropriate sexual satisfaction amongst couples. An excerpt of the definition derived from these texts suggests that a youth in sound health taking regularly some sort of Vajikarana remedy may enjoy the pleasure of youth every night during all the seasons of the year [8]. Old men, wishing to enjoy sexual pleasure or to secure the affections of women, as well as those suffering from senile decay or sexual incapacity, and persons weakened with sexual excesses may also use Vajikaran remedies. They are highly beneficial to handsome and opulent youths and to persons who have got many wives. According to Rasendra Sara Sangrah an ayurvedic text Vajikaran remedy makes a man sexually as strong as a horse (Vaji) and enables him to cheerfully satisfy the heat and amorous ardours of young maidens (Figure 1) [9, 10]. Though in scientific terms these claims may represent a populous outlook, the popularity of Vajikaran in Ayurvedic system of medicine is nonetheless undisputed with numerous claims and textual references made to them during the course of human history.

## 4. Sexual Functions: An Ayurvedic Overview

The sexual inadequacies discussed in Ayurveda are of the following six types:

1. A cessation of the sexual desire owing to the rising of bitter thoughts of recollection in the mind of a man, or a forced intercourse with a disagreeable woman (who fails to sufficiently rouse up the sexual desire in the heart of her mate) illustrates an instance of mental impotency.
2. Excessive use of articles of pungent, acid, or saline taste, or of heat making articles of fare leads to the loss of the Saumya Dhatu (watery principle) of the organism. This is another kind of impotency.
3. Virile impotency resulting from the loss of semen in persons addicted to excessive sexual pleasure without using any aphrodisiac remedy is the merit form of the virile impotency.
4. A long-standing disease of the male generative organ (syphilis, etc.) or the destruction of a local Marma such as the spermatic cord destroys the power of coition altogether.
5. Sexual incapacity from the very birth is called the congenital (Sahaja) impotency.
6. Voluntary suppression of the sexual desire by a strong man observing perfect continence or through utter apathy produces a hardness of the spermatic fluid and is the cause of the sixth form of virile impotence.

Of the six foregoing types of impotency, the congenital form as well as the one due to the destruction of any local Marma (spermatic cord) should be regarded as incurable, the rest being curable and amenable to the measures and remedies antidotal to their respective originating causes [11].

## 5. Ayurveda and the Concept of Aphrodisiacs

Traditional Ayurvedic treatise classified aphrodisiac in the following five categories, a few plants have been provided as references for each kind of the therapeutic class defined [3].

- Drugs which increase the quantity of semen or stimulate the production of semen for example,* Microstylis wallichii, Roscoea procera, Polygonatum verticillatum, Mucuna pruriens*, and* Asparagus racemosus*.
- Drugs which purify and improve the quality of semen for example,* Saussurea lappa, Myrica nagi, Sesamum indicum, Vetiveria zizanioides*, and* Anthocephalus cadamba*.
- Drugs which improve ejaculatory functions for example,* Strychnos nux vomica, Cannabis sativa, Myristica fragrans*, and* Cassia occidentalis*.
- Drugs delaying the time of ejaculation or improving ejaculatory performance for example,* Sida cordifolia, Asparagus racemosus, Cinnamomum tamala, Anacyclus pyrethrum, Mucuna pruriens, and Cannabis sativum*.
- Drugs arousing sexual desire, namely,* Withania somnifera, Asparagus racemosus, Datura stramonium, Anacyclus pyrethrum, Hibiscus abelmoschus*, and* Opium*.

Having discussed the Ayurvedic basis for the role of Vajikarana herbs, it is important to understand the role of modern pharmacology and an insight into the control of the sexual behavior in the human body.

## 6. Mechanism of Sexual Behavior: Modern Perspective

Our understanding of the process and initiation of sexual arousal is finding a more lucid basis, which stems from evidences in both preclinical and clinical studies. Sexual arousal is dependent on neural (sensory and cognitive), hormonal, and genetic factors, something also defined in Ayurveda as well but using a scientific language pertinent to this age.

## 7. Brain and Neurochemical Basis of Sexual Behavior

Drugs affecting sexuality can either act on the central nervous system (Brain) and/or on the peripheral nervous system. Drugs affecting the brain and presumably sex centers are generally attributed with an increase or decrease in sexual arousal. Drugs that affect peripheral nerves will not affect arousal directly but may affect sexual function. In some cases, drugs action is direct and involves chemical alteration of the neurons, which governs sexual arousal or function. Alternatively, some drugs may act indirectly by altering blood flow to the genitalia. Most hypotheses concerning the neurochemical basis of sexual behavior are derived from studies in animals, but in some cases support has been provided by clinical studies. Five major neurochemically distinct systems are supposed to work together for increasing sexual arousal. The transmitters include norepinephrine, dopamine, serotonin, acetylcholine, and histamine [12]. The most widely endorsed hypotheses suggest that both serotonin and dopamine are involved in the neurochemical control of sexual behavior with serotonin playing an inhibitory role and dopamine an excitatory role. Dopamine plays a crucial role in the central control of sexual behavior in males [13]. Increase in the activity of central dopaminergic systems correlates with sexual activity [14].* In vivo* microdialysis in conscious male rats revealed that dopamine transmission increases sharply in the striatum, nucleus accumbens, and medial preoptic area during copulation [15–17]. This change in central neurotransmission may be permissive to a series of motor responses including penile erection. It may also modulate the activity of brain nuclei directly involved in the control of penile erection [18]. For example, drugs such as levodopa, which increase levels of dopamine in the brain, tend to be associated with increase libido and enhanced sexual function in patients suffering from abnormal dopamine activity such as that associated with Parkinson's disease. In contrast, drugs blocking dopamine function such as haloperidol cause loss of sexual arousal. It has been long suspected that monoamines play a crucial role in the regulation of sexual behaviour, particularly that of dopaminergic transmission which is facilitatory to masculine activity and both dopaminergic and adrenergic receptors are involved. Yohimbine, bromocriptine, and reserpine are alpha-adrenergic receptor blocking agents whereas yohimbine, bromocriptine, amphetamine, and apomorphine all comet with the neurotransmitter dopamine for binding to membrane sites [19]. Furthermore, some studies have also suggested that the dopamine release is also increased during sexual activity in the paraventricular nucleus of the hypothalamus and that in this hypothalamic nucleus dopamine facilitates penile erection and sexual behaviour by activating NO production in the cell bodies of oxytocin neurons controlling penile erection and sexual motivation, which project to extrahypothalamic brain areas and to the spinal cord [13, 20–24]. Therefore, there appears to be a lot of cross talk at different neuronal levels between dopamine and nitric oxide; this has been discussed further in the next section.

## 8. Nitric Oxide-Based Mechanism of Sexual Behavior

Nitric oxide (NO) is an atypical regulatory molecule having the dual role as a secondary messenger/neurotransmitter. It has been implicated in diverse physiological functions [22]. Findings so far indicate that NO may also be a major neuronal messenger [23]. In particular, it is an established physiological mediator of penile erection [24] and in the brain; NO synthase is highly concentrated in structures directly or indirectly involved in sexual behavior (olfactory bulb, supraoptic and paraventricular nuclei, amygdala, septal structures, etc.) [25].

Recent studies suggest that NO is a major physiological stimulus for relaxation of penile vasculature and trabecular smooth muscle, essential for penile erection [26]. Relaxation of the trabecular smooth muscle of the corpus cavernosa leads to a decreased vascular resistance and increased blood flow to the penis. Alongside the increased flow, venous outflow is reduced by the compression of the subtunical venules. The combination of increased inflow and decreased outflow causes penile engorgement and erection. NO from the vascular endothelium of the sinusoids and from the nonadrenergic, noncholinergic, and cavernosal nerves appears to mediate the vasodilatation [27, 28]. The new drug used for the treatment of erectile dysfunction, and sildenafil acts by potentiating the effect of NO by inhibiting the specific enzyme phosphodiesterase-V that terminates the action of NO generated cGMP in the penile vasculature [29]. Many medicinal herbs and drugs derived from these herbs have been shown to have effects on the NO signaling pathway. For example, the saponins from ginseng (ginsenosides) have been shown to relax blood vessels (probably contributing to the antifatigue and blood pressure-lowering effects of ginseng) and corpus cavernosum (thus, for the treatment of men suffering from erectile dysfunction; however, the legendary aphrodisiac effect of ginseng may be an overstatement) [30].

## 9. Androgen-Based Mechanism of Sexual Behavior

Androgens play a crucial role in the development of secondary male sexual organs such as the epididymis, vas deferens, seminal vesicle, prostate, and the penis. Furthermore, androgens are needed for puberty, male fertility, and male sexual function [29]. Testosterone is the principal androgen secreted by the testes. Testosterone is synthesized in the Leydig cells of the testes, stimulated by luteinizing hormone (LH). One of the principal effects of testosterone within the testes is the stimulation of spermatogenesis in seminiferous tubules. The testosterone- or dihydrotestosterone-receptor complex next crosses the nuclear membrane, binds to DNA, and stimulates new mRNA synthesis and, thereby, new protein synthesis. The effect of testosterone on libido may require conversion of testosterone to estradiol in the hypothalamus. The mechanisms whereby testosterone affects muscle, bone, and the erythron do not appear to require prior molecular conversion [30].

Drugs used to treat various sexual problems are found to modify the action of neurotransmitters which could be facilitatory, inhibitory, or both. Androgens are known to influence NO production in the brain as well as in the periphery [31, 32]. NO is synthesized by the enzyme nitric oxide synthase (NOS) which plays an important role in many brain functions. NO function as a neurotransmitter and NOS is present in the regions of the brain that regulate sexual functions [33]. Interestingly, administration of testosterone to castrated male rats increases the number of NO synthase-labelled neurons in the mPOA, indicating an increase in NO synthesis [34]. NO is capable of stimulating dopamine (DA) release in the mPOA, which in turn stimulates penile erection. This mechanism may constitute one way in which androgens stimulate sexual arousal [35].

## 10. A Few Medicinal Herbs with Validated Effects on Sexual Functions

In the present section we would discuss a few of the many well-tested Ayurvedic and other traditional herbs, which have a long standing reputation as a cure for sexual dysfunction and which have been used in numerous preparations for improving sexual performance and fertility especially in case of males. Apart from these herbs large numbers of plants have also been tested and evaluated for effect on sexual functions and reproductive parameters, a comprehensive description and names of these herbs are provided in Table 2. Many researchers have investigated the active bioconstituent present in different herbs that are responsible for enhancing sexual activity, spermatogenesis and showing other positive effect in reproductive parameters (Table 3).

### 10.1. *Butea superba*

*Butea superba* Roxb (Leguminosae) is commonly found in Thai deciduous forests and has the domestic name of “Red Kwao Krua.” The plant tubers have long been consumed as a traditional medicine for the promotion of male sexual vigor.* B. superba* alcoholic extract (0.01, 0.1 or 1.0 mg/kg BW/day) for 6 months treatment significantly increased the sperm concentration and delayed the decreased motility with time. None of signs of sperm anomalies and testicular damages were observed [36]. Subchronic treatment of* B. superba* tuberous powder suspension at high doses (200 mg/kg) in male rats exhibited adverse effects to blood chemistry, haematology, and blood testosterone level. Powdered crude drug at the doses of 2, 25, 250, and 1250 mg/kg body weight was administered for 8 weeks; there was an increased testis weight and sperm counts in rat. Hematology as well as the liver and kidney function of all treated groups showed no difference from the control [37]. A dose-dependent decrease of only blood testosterone, but not LH, was significantly different from the control in the rats treated with high doses of plant powder. This present study suggests that testosterone disruption is significant, at least after 90 days of consumption of high doses of* B. superba* powder [38]. The ethanol extract of* B. superba* is effective in enhancing penile erection. The ethanol extract increased intracavernous pressure (ICP)* in vivo*. It also significantly enhanced the effects of cGMP and isobutylmethylxanthine. This suggests that* B. superba* may act through cAMP/cGMP pathways [39].

*Clinical Studies.* The plant powder showed potential activity in a human clinical trial for treatment of erectile dysfunction in males [40].

### 10.2. *Curculigo orchioides*

*Curculigo orchioides* Gaertn (Amaryllidaceae), also known as Kali Musli or Syah (black) Musli, is considered as aphrodisiac and Rasayan or rejuvenator [41]. The ethanolic extract of rhizome improved sexual behaviour in male rats. The sexual performance as assessed by determining parameters such as penile erection, mating performance, and sexual and orientation behavior was significantly improved. Moreover a pronounced anabolic and spermatogenic effect was evidenced by weight gains of reproductive organs. The treatment also markedly affected sexual behavior of animals as reflected in reduction of mount latency, an increase in mount frequency and enhanced attractability towards female. Penile erection index was also incremented in treated group [42, 43]. The lyophilized aqueous extracts of* Curculigo orchioides* significantly improved the pendiculatory activity in male rats after 14 days of treatment. Similarly, the extract could also preserve the* in vitro* sperm count when compared to control group after 30 min. of incubation [44]. The aqueous extract of the plant showed prominent activity at a dose level of 200 mg/kg. In general, a pronounced anabolic effect in treated animals was evidenced by weight gains in the body and reproductive organs. There was a significant variation in the sexual behavior of animals as reflected by reduction of mount latency, ejaculation latency, postejaculatory latency, intromission latency, and an increase of mount frequency. Penile erection was also considerably enhanced. Reduced hesitation time (an indicator of attraction towards female in treated rats) also indicated an improvement in sexual behavior of extract treated animals [45]. In case of physically induced sexual dysfunction, that is, heat induced damaged to the testicular function, the plant was useful in ameliorating the reduced spermatogenesis and the treated animals could effectively overcome the heat shock protein; this exemplified the role of* C. orchioides* in overcoming physically induced sexual dysfunction due to testicular damage [46].

### 10.3. *Cynomorium coccineum*

*Cynomorium coccineum* Linn. (Cynomoraceae) is known as Som-El-Ferakh in Saudi Arabia, which is a black leafless parasitic plant devoid of chlorophyll. The natives in Qatar use it (mainly with honey) as a tonic and aphrodisiac [47]. Aqueous extract of* Cynomorium coccineum* induced significant increase in the sperm count, improved the percentage of live sperm and their motility, and decreased the number of abnormal sperm. Testicular histology showed increased spermatogenesis and seminiferous tubules full of sperm in the treated group compared to the untreated controls [48]. Aqueous extract of the plant elicited notable spermatogenesis in immature rats. Serum testosterone and FSH levels were lower in animals treated with extracts than controls, whereas interstial cell stimulating hormone levels was higher in treated animals [49].

### 10.4. *Chlorophytum borivilianum*

Safed Musli (*Chlorophytum borivilianum*) belongs to the family Liliaceae with folkloric claims as aphrodisiac and sexual stimulant [50]. Ethanolic extract of roots as well as sapogenins isolated from the roots were studied for effect on sexual behavior and spermatogenesis in albino rats. Treatment had pronounced anabolic and spermatogenic effect in treated animals, evidenced by weight gains of body and reproductive organs. Administration of extracts markedly affected sexual behavior of animals reflected in reduction of mount ejaculation, postejaculatory, and intromission latency. An increase in mount frequency and attractability towards female was observed [51]. The aqueous extract of dried roots of* Chlorophytum borivilianum* enhances the sexual arousal, vigor, and libido in Wistar rats. The extract increases sperm count significantly [44, 52].

In case of streptozotocin and alloxan induced hyperglycemia, the aqueous extract from the plant resulted in amelioration of sexual dysfunction, resulted in improved sexual performance compared to diabetic control rats. The study thus provided evidence that herbal drugs may act on sexual dysfunction in normal as well as diabetic animals [53, 54].

### 10.5. *Epimedium koreanum*

The traditional Chinese medicinal herb,* Epimedium* L. (Berberidaceae), is a popular botanical supplement used as a health tonic. Most important* Epimedium* species used for medicinal purposes are* E. koreanum* Nakai,* E. pubescens* Maxim.,* E. brevicornum* Maxim,* E. sagittatum* (Sieb. Et Zucc) Maxim, and* E. wushanense* T.S. Ying [55]. Hydroalcholic extract of the plants are reputed to produce aphrodisiac effects and are commonly used in Chinese herbal medicine to enhance erectile function [56]. It is thought that icariin is likely to be the primary active component of* Epimedium* extracts. Icariin is a flavonol, a type of flavonoid. It is the prenylacetylation of kaempferide 3,7-O-diglycoside, icariin on erectile dysfunction and established its dose-dependent selective inhibitory effect on phosphodiesterase-5 (PDE5). Oral treatment with icariin (>98.6% purity) for 4 weeks potentially improves erectile function. This effect is correlated with an increase in the percentage of smooth muscle and the expression of certain NO synthase isoforms in the corpus cavernosum of castrated rats. These results suggest that icariin may have a therapeutic effect on erectile dysfunction [57]. Icariin was inhibitory to all three PDE5 isoforms and with similar IC50 values, which were approximately three times greater than those for zaprinast. Icariin was able to enhance the levels of cyclic guanosine monophosphate in sodium nitroprusside-treated cavernous smooth muscle cells [58–60] and to enhance the production of bioactive nitric oxide [61] as well as mimicking the effects of testosterone [62].

### 10.6. *Eurycoma longifolia*

*Eurycoma longifolia* Jack (Simaroubaceace), known locally as Tongkat Ali, is commonly found in lowland forests. It is very commonly used by ethnic groups for numerous reasons and is one of the major export components from Malaysia [63].* E. longifolia* increases sexual motivation in sexually naive male rats. An electric grid was used as an obstruction in the electrical copulation cage in order to determine how much an aversive stimulus the sexually naive male rat for both the treated with* E. longifolia* and control groups were willing to overcome to reach the estrous receptive female in the goal cage. The intensity of the grid current was maintained at 0.12 mA and this was the intensity in which the male rats in the control group failed to crossover to reach the goal cage. Results showed that* E. longifolia* Jack continued to enhance and also maintain a high level of both the total number of successful crossovers, mountings, intromissions, and ejaculations during the 9–12th week observation period [64]. Ethanol extract treatment for 10 days increased the sexual performance of the treated male rats by extending the duration of coitus and decreasing the refractory period between the different series of copulation [65]. Administration of 800 mg/kg of butanol, methanol, water, and chloroform fractions of* E. longifolia* significantly increased the leavator ani muscle when compared with the control (untreated) in the uncastrated intact male rats and when compared to control (untreated) in the testosterone-stimulated castrated intact male rats [66].* E. longifolia* continued to enhance and also maintain a high level of both the total number of successful crossovers, mountings, intromissions, and ejaculations during the 9–12th week observation period. Butanol, methanol, water, and chloroform extracts of the roots of* E. longifolia* produced a dose-dependent, recurrent, and significant increase in the episodes of penile reflexes as evidenced by increases in quick flips, long flips, and erections of the treated male rats during the 30-min observation period [67].* E. longifolia* (0.5 g/kg) for three-week increase in the percentage of the male rats responding to the right choice, more than 50% of the male rats scored “right choice” after 3 weeks posttreatment and the effect became more prominent after 8 weeks posttreatment (only 40–50% of the control male rats responded to the right choice) using the electrical copulation cage [68]. The middle-aged male rats treated with 800 mg/kg of* E. longifolia* increased orientation activities towards the receptive females as evidenced by increase in anogenital investigatory behaviour, licking, and mounting but possessed a lack of interest in the external environment as evidenced by decrease in climbing, raring, and exploration on the caged wall, as well as it also enhanced self-orientation as evidenced by increased grooming of their own genitals and also showed restricted confinement, with targeted orientation and movement toward female as compared with the controls; it also enhanced the sexual qualities of the middle-aged male rats by decreasing their hesitation time as compared to controls with various fractions of* E. longifolia* [69, 70].

### 10.7. *Lepidium meyenii*

*Lepidum meyenii* Walp (Brassicaceae) known as Maca is the edible root traditionally employed for its purported aphrodisiac and fertility-enhancing properties. Subacute oral administration of hexanic, methanolic, and chloroform extracts of Maca (*Lepidium meyenii*) root significantly decreased intromission latency and intercopulatory interval and increased intromission frequency and copulatory efficacy as compared to controls. Hexanic and methanolic extracts were able to increase mount frequency, while only hexanic fraction significantly improved mount latency. Globally, only the hexanic fraction significantly improved the majority of the sexual parameters measured. Subacute oral administration of hexanic Maca extract improved sexual performance parameters in sexually inexperienced male rats most effectively [71]. Oral administration of lipidic extract from* Lepidium meyenii* enhanced the sexual function of the mice and rats, as evidenced by an increase in the number of complete intromissions and the number of sperm-positive females in normal mice, and a decrease in the latent period of erection in male rats with erectile dysfunction [72]. Improvement of larginine-nitric oxide activity has also been attributed to Maca. The acute and daily administration of Maca in sexually experienced male rats produced a small change in ejaculation latency and postejaculatory interval and these changes disappeared with chronic treatment. Chronic administration of Maca did not increase anxiety and had some effect on locomotor activity [73]. Black maca appeared to have more beneficial effects on sperm counts and epididymal sperm motility after 42 days of treatment [74]. Maca has also shown effectiveness as a lead treatment for the dysfunction arising due to metalic lead exposure. Maca protects spermatogenesis by increasing lengths of stages VIII and IX–XI and daily sperm count that result in an increase in epididymal sperm number [75]. Oral treatment with ethyl acetate fraction of the hydroalcoholic extract of Black Maca for 7 days had the most beneficial effect on epididymal sperm count and daily sperm count compared with other fractions [76].

*Clinical Studies.* Maca enhanced fertility in both men and women [77, 78]. Improvement of sexual desire is not related to changes in pituitary or gonadal hormones [79, 80]. Maca does not activate androgen receptors and may actually block androgen receptors [81, 82]. Maca aqueous extract can be considered safe in doses up to 5 g extract/kg, corresponding to some 11 g dry hypocotyls/kg. The effect on reproductive physiology may be observed at 0.10 g extract/kg of Maca extract that represents 15.4 g of dry hypocotyls for an individual of 70 kg [83].

### 10.8. *Mucuna pruriens*

*Mucuna pruriens* Linn. Family Leguminosae is a popular Indian medicinal plant, which has long been used in traditional Ayurvedic Indian medicine. The total alkaloids from the seeds of* M. pruriens* were found to increase spermatogenesis and weight of the testes, seminal vesicles, and prostate in the albino rat [84].* M. pruriens* stimulated sexual function in normal male rats which was observed by increase in mounting frequency, intromission frequency, and ejaculation latency [85].* M. pruriens* seed powder improved significantly various sexual parameters copulatory behavior including mount frequency, mount latency, intromission frequency, and intromission latency of the male albino rats [86]. The ethanolic extracts of* M. pruriens* seed produced a significant and sustained increase in the sexual activity of normal male rats at a particular dose (200 mg/kg). There is significantly increased mounting frequency, intromission frequency, and ejaculation latency and decreased mounting latency, intromission latency, postejaculatory interval, and interintromission interval [87].* M. pruriens* efficiently recovered the spermatogenic loss induced due to ethinyl estradiol administration to rats. The recovery is mediated by reduction in ROS level, restoration of MMP, regulation of apoptosis, and eventual increase in the number of germ cells and regulation of apoptosis. The major constituent L-DOPA of* M. pruriens* largely accounts for prospermatogenic properties [88]. Administered of seed extract of* M. pruriens* to diabetic rats showed significant improvement in sexual behavior, libido and potency, sperm parameters, DSP, and hormonal levels as compared to diabetic rat without extract treatment [89].

*Clinical Studies.* The Treatment with* M. pruriens* seeds increased sperm concentration and motility in all the infertile study groups in man. After the treatment of extract the seminal plasma of all the infertile groups, the levels of lipids, antioxidant vitamins, and corrected fructose were recovered after a decrease in lipid peroxides after treatment Their was recovered sperm concentration significantly in oligo-zoospermic patients, but sperm motility was not restored to normal levels in astheno-zoospermic men [90].* M. pruriens* significantly improved T, LH, dopamine, adrenaline, and noradrenaline levels and reduced levels of FSH and PRL in infertile men. It also significantly recovered sperm count and motility.* M. pruriens* treatment to infertile men regulates steroidogenesis and improves semen quality [91, 92]. Treatment with* M. pruriens* significantly inhibited lipid peroxidation, elevated spermatogenesis, and improved sperm motility of infertile male and also improved the levels of total lipids, triglycerides, cholesterol, phospholipids, and vitamin A, C, and E and corrected fructose in seminal plasma of infertile men [93].* M. pruriens* significantly ameliorated psychological stress and seminal plasma lipid peroxide levels along with improved sperm count and motility. Treatment also restored the levels of SOD, catalase, GSH, and ascorbic acid in seminal plasma of infertile men. It reactivates the antioxidant defense system of infertile men and also helps in the management of stress and improves semen quality [94].

### 10.9. *Tribulus terrestris*

The plant* Tribulus terrestris* Linn. (Zygophyllaceae) popularly known as puncture vine is a perennial creeping herb with a worldwide distribution. Since ancient times it is regarded as an aphrodisiac in addition to its beneficial claims on various ailments such as urinary infections, inflammations, leucorrhoea, oedema, and ascites [95].* T. terrestris* has long been used in the traditional Chinese and Indian systems of medicine for the treatment of various ailments and is popularly claimed to improve sexual functions. Administration of* T. terrestris* to male lambs and rams improves plasma testosterone and spermatogenesis [96]. It also found to increase the levels of testosterone, luteinizing hormone [97], dehydroepiandrosterone, dihydrotestosterone, and dehydroepiandrosterone sulphate [98, 99]. The corpus cavernosal tissues obtained from New Zealand white rabbits following treatment with* T. terrestris* were tested* in vitro* with various pharmacological agents and electrical field stimulation and was found to have a proerectile effect [100].* T. terrestris* has been found to increase sexual behaviour in rats. Treatment of castrated rats with* T. terrestris* extract showed increase in prostate weight and intracavernosal pressure. There was an improvement of the sexual behaviour parameters as evidenced by increase in mount frequency and intromission frequency; decrease in mount latency, intromission latency, and penile erection index [101, 102].* T. terrestris* administration in rats increased the NADPH-d positive neurons and androgen receptor immunoreactivity in the PVN region. Androgens are known to increase both androgen receptor and NADPH-d positive neurons either directly or by its conversion to oestrogen. The mechanism for the observed increase in AR and NADPH-d positive neurons in the present study is probably due to the androgen increasing property of* T. terrestris* [103].* T. terrestris* also increased the synthesis of cyclic nucleotides in CCSM cells [101].* T. terrestris* extract increased the levels of T, DHT, and DHEAS and that the effect was more pronounced in hypogonadal state. Such increase in androgen levels could be the responsible factor for the age-old claims of PTN as an aphrodisiac and therefore* T. terrestris* may be useful as an adjunct in mild to moderate cases of ED [104]. The ability of tribulus to increase the release of nitric oxide may account for its claims as an aphrodisiac [100, 102].

### 10.10. *Withania somnifera*

Ashwagandha (*Withania somnifera* (L.) Dunal, Family: Solanaceae) is also known as Indian ginseng commonly used in Ayurvedic medicine. It is best regarded as adaptogen, tonic with aphrodisiac properties. Some workers have reported the decrease mating behavior and antifertility effects of* W. somnifera* root on in mice [105]. The root extract induced a marked impairment in libido, sexual performance, sexual vigour, and penile erectile dysfunction [106]. It also showed antifertility activity in male rat [107]. But some scientist shows that* W. somnifera* has the capability of combating stress-induced infertility. It also protects swimming-induced reproductive endocrine dysfunctions in male rat [108]. Aqueous extract improved spermatogenesis, which may be due to increased interstitial cell stimulating hormone and testosterone-like effects as well as the induction of nitric oxide synthase [109].

*Clinical Studies.* Ashwagandha root extract administered to the oligospermic patients resulted in a significantly greater improvement in spermatogenic activity and serum hormone levels as compared to the placebo treated [110]. Treatment of infertile men with* Withania somnifera* inhibited lipid peroxidation and protein carbonyl content and improved sperm count and motility. It also recovered the seminal plasma levels of antioxidant enzymes* W. somnifera* root powder when administered in a dosage of 5 g/day for 3 months to normozoospermic infertile man resulted in a decrease in stress, improved the level of antioxidants, and improved overall semen and vitamins A, C, and E and corrected fructose. Significantly increased serum T and LH and reduced levels of FSH and PRL in infertile men were observed [111, 112].

## 11. Conclusion

Various herbs have been used by people of different cultures to treat conditions of male infertility or for treatment of reproductive disorders. They have also been advocated for improving sexual desire as well as sexual performance and erectile dysfunction, vasodilatation, increased testosterone level, brain monoamines, effect on pituitary-gonadal axis, and so forth are suggested mechanism for its action of these herbs [113].

In absence of clinical efficacy and safety data on these herbs, people are skeptical to use them. There is an urgent need to conduct clinical studies to support traditional claims and to work out cellular and molecular mechanism involved. Investigations in validation of the herbs will go a long way in management of infertility. Moreover, the cross talk of various pathways involved must also be taken into account to come up with a molecular pathway to find a lead molecule of herbal origin of the treatment of various forms of sexual dysfunction.

## Figures and Tables

**Table 1:** List of plant reported in Ayurveda as Vajikaran Rasayan.

Serial number	Hindi name	Botanical name	Family	Parts use	Uses
1	Akarkara	*Anacyclus pyrethrum *DC	Asteraceae	Dried roots	Vajikaran, Balakarka
2	Akharot	*Juglans regia *Linn.	Juglandaceae	Dried cotylcdous	Vrsya, Bala, Sukral
3	Adarakha	*Zingiber officinalis *Rosc.	Zingiberaceae	Fresh rhizomes	Vrsya
4	Bhrngaraja	*Elcipta alba *nassle	Asteraceae	Whole plant	Balya, Rasayana
5	Manduka parni	*Bacopa monnieri *Linn.	Scrophularaceae	Dried whole plant	Rasayana
6	Anar	*Punica granatum *Linn.	Punicaceae	Dried seed	Sukralya, Balya
7	Gambhari	*Gmeline arborea *Roxb.	Verbenaceae	Dried fruit	Rasayana, Sukrala
8	Ganna	*Saccharum officinarum *Linn.	Podceae	Dried stem	Vrsya, Balya
9	Jayata	*Sesbana sesbanl *Linn.	Fabaceae	Fresh & dried root	Rasayana
10	Talmakhana	*Asteracantha longifolia *Nees	Acanthaceae	Whole plant seed	Baiya, Vrsya, Vajikarna
11	Makoya	*Solanum nigrum *Linn.	Solanaceae	Dried whole plant	Rasayana, Vrsya
12	Kaitha	*Feronia limonia *Linn.	Rutaceae	Dried pulp of mature fruit	Vrsya
13	Mahuwa	*Madhuca indica *	Saptoceae	Flower	Sukrala, Balya
14	Tesu	*Butea monosperma *Lam.	Fabaceae	Dried stem bark	Vrsya
15	Gandha prasarini	*Paederia foetida *Linn.	Rubiaceae	Whole plant	Vrsya
16	Piyal	*Buchanania lanzan *Spreng	Anacardiaceae	Seed	Vrsya, Bala
17	Chaval	*Oryza sativa *Linn.	Poaceae	Dried root	Balya, Rasayana
18	Shankhapusphi	*Convolvulus pluricaulis *Chois	Convolulacea	Whole plant	Balya, Rasayana
19	Vidari kanda	*Pueraria tuberosa *DC	Leguminosae	Sliced & dried pieces of tuberous root	Sukralya, Balya, Rasayana
20	Basanaay	*Aconitum Chasmanthum *	Ranunculaceae	Dried roots	Rasayana
21	Jav	*Hordeum vulgare *Linn.	Poaceceae	Dried fruit	Vrsya, Balya
22	Amla	*Emblica officinalis *	Euphorbiaceae	Fresh fruit pulp	Vrsya, Rasayna
23	Vijayasara	*Pterocarpus marsupium *Roxb.	Leguminosae	Heart wood	Rasayna
24	Asagandha	*Withania somnifera *Dunal	Solanaceae	Dried mature roots	Vajikarana, Balya, Rasayana
25	Kunghi	*Abutilon indicum *Linn.	Malvaceae	Roots	Balya, Vrsya
26	Bela	*Aegle marmelos *	Rutaceaeae	Ripe fruit	Balya
27	Gokhru	*Tribulus terrestris *Linn.	Zygophyllaceae	Root fruit	Vrsya
28	Giloe	*Tinospora Cordifolia *	Menispermaceae	Stem	Balya, Rasayana
29	Gugal	*Commiphora wightii *	Burseraceae	Exudate	Balya
30	Harad	*Terminallia chebula *Retz	Combretaceae	Mature fruit	Rasayana
31	Jaiphal	*Myristica fragraus *	Myristicaceae	Dried seeds	Vrsya
32	Kapasa	*Gossypium herbaceum *Linn.	Malvaceae	Seed	Vrsya
33	Kasesu	*Scirpus kysoor *Roxb.	Cyperaceae	Rhizome	Sukra
34	Kerada	*Pandanus tectorius *sokmel	Pandanaceae	Root	Balya, Rasayana
35	Saunt	*Foeniculum Vulgare *Mill	Umbelliferae	Ripe fruit	Balya
36	Bhaang	*Cannabis sativa *Linn.	Cannabaceae	Dried leaves	Vajikara
37	Mulethi	*Glycyrrhiza glabra *Linn.	Leguminosae	Root	Balya, Vrsya
38	Hadjod	*Cissus quadrangularis *Linn.	Vitaceae	Dried stem	Vrsya
39	Kewandr	*Mucuna prurita *Hook.	Fabaceae	Mature seed	Balya, Vrsya
40	Munkka	*Vitis vinifera *Linn.	Vitaceae	Dried mature fruit	Vrsya
41	Evana	*Ricinus communis *Linn.	Euphorbiaceae	Fresh leaf	Vrsya
42	Bichu hathjori	*Martynia annua *Linn.	Martyniaceae	Dried seed	Rasayan
43	Kakoli	*Lillum polyphyllum *D.Don	Liliaceae	Tuberous root	Sukrala
44	Kamal kand	*Nelumbo nucifera Gaertn *	Nymphaeaceae	Rhizome	Vrsya
45	Kasa	*Saccharum spontaneum *Linn.	Poaceae	Root stock	Vrsya, Bala
46	Kui	*Nymphaea alba *Linn.	Nymphaeaceae	Dried flowers	Balya
47	Lahasun	*Allium sativum *Linn.	Liliaceae	Bulb	Balya, Vrsya, Rasayna
48	Pitabala	*Sida rhomifolia *Linn.	Malvaceae	Dried root	Sukra, Balya
49	Manjitha	*Rubia cordifolia *Linn.	Rubiaceae	Stem	Vrsya, Rasanyana
50	Mashvan	*Teramnus iabialis *Spreny	Fabaceae	Whole plant	Balya, Vrsya
51	Masur	*Lens culinaris *medic	Fabaceae	Dried seeds	Balya
52	Pan	*Piper betle *Linn.	Piperaceae	Leaf	Balya, Vrsya
53	Nariyal	*Cocos nucifera *Linn.	Arecaceae	Dried endosperm	Balya, Vrsya
54	Rakta chandana	*Petrocarpus santalinus *Linn.	Fabaceae	Heard wood	Balya, Vrsya
55	Sarivan	*Desmodium gangetium DC *	Fabaceae	Dried root	Balya, Vrsya
56	Chaval	*Oryza sativa *Linn.	Poaceae	Dried fruit	Vrsya
57	Sarkand	*Saccharum bengalense *Retz.	Poaceae	Root	Balya, Vrsya
58	Gulab	*Rosa centifolia *Linn.	Rosaceae	Dried flower	Sukra
59	Seesam	*Dalbergia sissoo *Roxb.	Fabaceae	Stem bark	Balya
60	Jhuner	*Taxus baccata *Linn.	Taxaceae	Dried leaf	Sukravarahake
61	Safed chandan	*Santalum album *Linn.	Santalaceae	Heart wood	Vrsya
62	Tal	*Borassus flabellifer *Linn.	Araceae	Male inflorescence	Vrsya
63	Louki	*Lagenaria siceraria *	Cucurbitaceae	Fresh fruit	Vrsya
64	Neel kanal	*Nymphaea stellata *Willd	Nymphaeaceae	Dried flower	Rasayana

The meanings of the Sanskrit words are Vrsya: increase sexual potential; Rasayan: that nourishes body, boosts immunity, and helps to keep the body and mind in best of health. Balya: that acts as vitilizer; Sukra: increase sperm count.

**Table 2:** A list of few of the various popular herbs with ehtnopharmacological backing for being used as aphrodisiac. The table also describes the possible mechanism of action of one or more constitutent isolated from them.

Serial number	Biological source	Part used	Extract used	Mechanism of action	References
1	*Aframomum Melegueta* Roscoe (*Zingiberaceae*)	Fruits	Aqueous extracts	Improvement of sexual behaviour	[114]
2	*Asparagus racemosus *Willd. (Liliaceae)	Roots	Aqueous extracts	Improvement of sexual behaviour	[115]
3	*Allium tuberosum *Rottl. ex Spreng (Liliaceae)	Seeds	Butanolic extract	Improvement of sexual behaviour	[116]
4	*Alpinia calcarata *Roscoe (Zingiberaceae)	Rhizomes	Hot Aqueous extract	Increase of sexual behaviour And testosterone	[117]
5	*Argeria nervosa syn. A. speciosa *Sweet (Convolvulaceae)	Fresh leaves, roots and flowers	Aqueous, ethanol, hexane extract	Improvement of sexual behaviour	[118]
6	*Aspidosperma ulei *Markgr. (Apocyanaceae)	Root	Alkaloidal rich fraction	Increase penile erection	[119]
7	*Asteracantha longifolia *Nees (Syn) (Acanthaceae)	Seeds	Ethanolic extract	Improvement of sexual behaviour	[120]
8	*Anacyclus pyrethrum *DC (Compositae)	Root	Aqueous extract, Ethanolic extract	Improvement of sexual behaviour	[121–123]
9	*Anacardium occidentale *L. (Anacardiaceae)	Leaf	Hexane extract	Increase fertility	[124]
10	*Bulbine natalensis *Baker (Asphodelaceae)	Stem	Aqueous extract	Increase hormone level	[125]
11	*Butea Frondosa *Koen. ex Roxb. (Leguminosae)	Bark	Aqueous extract	Improvement of sexual behaviour	[126]
12	*Butea superba *Roxb (Leguminosae)	Tuber	Ethanolic extracts	Increase penile erection	[39]
13	*Bryonia lacinosa *Linn (Cucurbitaceae)	Seeds	Ethanolic extracts	Improvement of sexual behaviour	[127]
14	*Basella alba* L. (Basellaceae)	Leaves	Terpenoid or steroid compounds	Increased the blood testosterone concentrations	[128]
15	*Boesenbergia rotunda* (L.) Mansf. (Zingiberaceae)	Rhizome	Ethanolic extract	Improvement of sexual behaviour	[129]
16	*Casimiroa edulis *La Llave (Rutaceae)	Seed	Aqueous extract	Improve sexual activity	[130]
17	*Camellia sinensis* (L.) O. Kuntze (Theaceae),	Leaves and buds	Aqueous extract	Increased the blood testosterone concentrations	[131]
18	*Catha edulis *Forsk (Celestreceae)	Shoots and Small branches	Chloroform : diethyether extract (1 : 3)	Improvement of sexual behaviour	[132]
19	*Caesalpinia benthamiana *L. (Caesalpiniaceae)	Roots	Aqueous extract	Improve sexual activity	[133]
20	*Chlorophytum borivilianum *Sant*. (*Liliaceae)	Roots	Ethanolic extract	Improve sexual activity	[51]
21	*Cocculus hirsutus* Linn (Menispermaceae)	Aerial part	Methanolic extract	Spermatogenic	[134]
22	*Curculigo orchioides *Gaertn (Amaryllidaceae)	Rhizome	Ethanolic extract	Improve sexual activity	[42, 135]
23	*Crocus sativus *L. (Iridaceae)	Stigma	Aqueous extract	Improve sexual activity	[136]
24	*Cynomorium coccineum* L. (Cynomoraceae)	Roots	Water Extract	Increased spermatogenesis and increase sperm count	[48]
25	*Diodia scandeus* SW*. (Rubiaceae) *	Herbs	Ethanolic extract	Potentiates the action of ACh and adrenaline	[137]
26	*Dracaena arborea* (Wild) Link (Dracaenaceae)	Root	Aqueous and Ethanolic	Improve sexual activity	[138]
27	*Dactylorhiza hatagirea (*D.Don) Soo (Orchidacea)	Tubers	Aqueous extract	Improve sexual activity	[139]
28	*Eurycoma longifolia *Jack. (Simaroubaceae)	Roots	Methanolic extracts	Improve sexual activity	[67]
29	*Eulophia nuda *Lind. (Orchidaceae)	Tubers	Powder	Improve sexual activity	[140]
30	*Epimedium koreanum *Nakai (Berberidaceae)	Herbs	Aqueous and Ethanolic	Improve sexual activity	[56]
31	*Epimedium brevicornum* Maxim (Berberidaceae)	Root	Aqueous Extract	Increase Nitric oxide release	[141]
32	*Fadogia agrestis *Schweinf. Ex Hiern (Rubiaceae)	Stem	Alkaloids and Saponins	Increased the blood testosterone concentrations	[142]
33	*Ferula hermonis *Boiss (Umbelliferae)	Roots	Acetonic extract	Stimulated sexual motivation	[143]
34	*Garcinia cambogia *Desr. (Clusiaceae)	Seeds	Biflavonoid and xanthone	Increase sperm count	[144]
35	*Hibiscus sabdariffa* L. (Malvaceae)	Flowers	Anthocyanins	Increase Sperm count	[145]
36	*Hibiscus macranthus* Hochst. Ex A.Rich. (Malvaceae)	Leaves	Terpenoid or steroid compounds	Increased the blood testosterone concentrations	[128]
37	*Kaempferia parviflora *Wall. Ex. Baker (Zingiberaceae)	Rhizomes	Alcohol extract	Increase in blood flow to the testis, Sexual behaviour	[146, 147]
38	*Lepidium meyenii* Walpers (Brassicaceae)	Root	Aqueous extract	Spermatogenesis	[83]
39	*Lophira laceolata* (Ohanacea)	Stem Bark	Aqueous extract	Improve sexual behaviour	[148]
40	*Lithospermum arvense *L. (Boraginaceae)	Seed	Aqueous extract	Androgenic	[149]
41	*Massularia acuminata* (G. Don) Bullock ex Hoyl. (Rubiaceae)	Stem	Aqueous extract	Stimulate male sexual maturation	[150]
42	*Mondia whitei* (Hook f.) Skeels. (Periplocaceae)	Roots	Aqueous extract	Increase in the testicular weight and serum and testicular testosterone	[151, 152]
43	*Mucuna pruriens *Baker (Fabaceae)	Seed	Chloroform extract	Spermatogenic	[84]
44	*Microdesmis keayana *J.Léonard. (Pandaceae)	Roots	Keayanidine B and keayanine	Stimulate NO production	[153]
45	*Montanoa tomentosa *Cerv (Asteraceae)	Leaves and flowers	Aqueous extract	Improve sexual behaviour	[154]
46	*Myristica fragrans* Houtt. (Myristicaceae)	Nutmeg	50% ethanolic extracts	Improve sexual behaviour	[155, 156]
47	*Orchis latifolia* Linn (Orchidaceae)	Roots	Aqueous extract, Powder	Improve sexual behaviour	[140, 157]
48	*Panax ginseng* C. A. Mayer (Araliaceae)	Roots	Ginsenosides, saponin glycosides	Increase Nitric oxide central nervous system action	[158]
49	*Panax quinquefolium* L. (Araliaceae)	Roots	Powdered	Facilitate male copulatory behavior	[159]
50	*Pausinystalia yohimbe* Pierre ex Beille (Rubiaceae)	Bark	Yohimbine	Improve sexual behaviour And penile erection	[160]
51	*Peganum harmala *L. (Zygophyllaceae)	Seed	Powdered	Improve semen quality, Spermatogenesis and organ weight	[161]
52	*Pentadiplandra brazzeana* Baill. (Capparidaceae)	Root	Aqueous extract	Serum and testicular testosterone levels testicular cholesterol, the seminal vesicular fructose	[162]
53	*Pfaffia paniculata* (Martius) Kuntze (***Amaranthaceae***)	Root	Aqueous extract	Increase serum testosterone	[163]
54	*Piper Gnineense Schumach. *& *Thonn. (Piperaceae) *	Fruits	Aqueous extract	Improvement of sexual behaviour	[114]
55	*Psidium guajava *Linn (Myrtaceae)	Leaves	Ethanol extract	Increase sperm count	[164]
56	*Psoralea corylifolia *L. (Fabaceae)	Fruits	Aqueous extract	Increased sperm counts, induces spermatogenesis	[165]
57	*Passiflora incarnate *Linn (Passifloraceae)	Leaves	Methanolic extract	Increase sexual behaviour	[166]
58	*Rubus coreanus *Miq (Rosaceae)	Fruit	Powder	Enhancing spermatogenesis	[167]
59	*Rhoicissus tridentata *L.f. (Wild and R.B. Drumm) (Vitaceae)	Root bark	Chloroform and Ethanolic extract	Relaxed the corpus cavernosal smooth muscle	[168]
60	*Syzygium aromaticum (L.) Merr. *&* Perry. *	Flower bud	50% ethanolic extract	Increase sexual behaviour	[169]
61	*Senecio cardiophyllus *Hemsl (Asteraceae)	Root	Aqueous extract	Increase the ejaculatory capacity	[170]
62	*Salvia haematodes *L. (Lamiaceae)	Roots	Ethanolic extract	Increase sexual behaviour	[171]
63	*Securidaca longepedunculata* (Fresen) (Polygalaceae),	Root bark	Chloroform and Ethanolic extract	Relaxed the corpus cavernosal smooth muscle	[168]
64	*Terminalia catappa *Linn. (Combretaceae)	Seeds	Seed suspension	Increase sexual behaviour	[172]
65	*Tribulus terrestris *L. * (Zygophyllaceae) *	Fruits	Protodioscin	Androgen increasing property	[101]
66	*Tribulus alatus* Delile (Zygophyllaceae)	Aerial parts and fruits	70% alcoholic extract	Increase serum testosterone	[173]
67	*Trichopus Zeylanicus* Gaertn (Trichopodaceae)	Leaf	Ethanolic extract	Stimulate sexual behaviour	[174]
68	*Tricholepis glaberrima. *DC (Compositae)	Aerial parts	Methanol extract	Stimulate sexual behaviour	[175]
69	*Turnera diffusa *Willd (Turneraceae)	Leaf	30% ethanol in water (v : v),	Stimulate sexual behaviour	[176]
70	*Vanda tessellate (*Roxb.) Hook. Ex Don (Orchidaceaae)	Root, flower	Aqueous and ethanolic	Stimulate sexual behaviour	[177]
71	*Wrightia natalensis* (Stapf) (Apocynaceae)	Root bark	Chloroform and Ethanolic extract	Relaxed the corpus cavernosal smooth muscle	[168]
72	*Withania somnifera* (L.) Dunal. (Solanaceae)	Root	Aqueous extract	Spermatogenesis	[49]
73	*Zingiber officinale* Roscoe (Zingiberaceae)	Roots	Aqueous extract	Increase of both testis weight and serum testosterone levels	[162]
74	Pueraria tuberosa DC (Fabaceae)	tubers	Ethanolic extract	Stimulate sexual behavior	[178]
75	Spilanthes acmella Murr. (Asteraceae)	flowers	Ethanolic extract	Stimulate sexual behavior	[179]
76	*Pedalium murex* Linn. (Pedaliaceae)	fruits	Ethanolic extract	Stimulate sexual behavior	[180]
77	*Dracaena arboreaI* (Willd.) (Asparagaceae)	Roots	Aqueous and ethanolic extracts	inhibit the activity of the bulbospongiosus muscles	[181]
78	*Allanblackia floribunda* Oliv. (Guttiferae)	Stem bark	Aqueous and ethanolic extracts	inhibit the activity of the bulbospongiosus muscles	[182]
79	*Corchorus depressus *Linn (Tiliaceae)	Whole plant	Chlorofrm fraction	Stimulate sexual behavior	[183]
80	*Arctium lappa *L (Asteraceae)	Root	Aqueous extract	Stimulate sexual behavior	[184]
81	*Musa paradisiacal *L (Musaceae)	Root	Aqueous extract	Increase testosterone level	[185]
82	*Lecaniodiscus cupanioides *Planch. (Sapindaceae)	Root	Aqueous extract	Increase testosterone level	[186]

**Table 3:** A tabular overview of some of the active consitutent with scientific findings and source description.

Serial number	Plant name	Active constituent	References
1	*Andrographis paniculata *Wall.ex Nees (Acanthaceae)	Andrographolide	[187]
2	*Aspidosperma ulei *Markgr (Apocyanaceae)	Alkaloidal fraction	[119]
3	*Catha edulis* (Vahl) Forssk. ex Endl. (Celastraceae)	Cathinone	[188]
4	*Crocus sativus *L. (Iridaceae)	Crocetin, Crocin	[136]
5	*Epimedium sagittatum* (Siebold & Zucc.) Maxim. (Berberidaceae)	Icariin	[58]
6	*Ferula hermonis *L. (Umbelliferae)	Ferutinin	[189, 190]
7	*Panax ginseng* L. (Araliaceae)	Ginsenosides	[158, 191]
8	*Lepedium meyeni *Walp (Oleacaceae)	Meiconoides	[71]
9	*Lycium barbaru *L. (Solanaceae)	Polysaccharides	[192]
10	*Mucuna macrocarpa* Wall. (Leguminosae)	Quercetin	[193]
11	*Mucuna pruriens *Baker. (Leguminosae)	Total Alkaloids	[84]
12	*Microdesmis keayana *J. Léonard (Pandaceae)	Keayanidine B and keayanine	[153]
13	*Palisota Hirsuta (*Thunb.) K. Schum. (Commelinaceae)	Total Flavonoids	[194]
14	*Pausinystalia yohimbe *Pierre ex Beille (Rubiaceae)	Yohimbine	[195, 196]
15	*Securidaca longepedunculata *Fres (Polygalaceae)	Novel xanthones,	[197]
16	*Satureja khuzestanica *Jamzad (Lamiaceae)	Essential oil;	[198]
17	*Tribulus terrestris *L. (Zygophyllaceae)	Saponins, Furostenol Glycoside	[100, 199]
18	*Turnera diffusa *Wild (Turneraceae)	Flavonoids	[200]
19	*Zingiber officinale *Roscoe (Zingiberaceae)	Zingerone, Gingerdiol	[145]
20	*Vanda tessellata* (Roxb.) ex Don (Orchidaceae)	2,7,7-tri methyl bicyclo [2.2.1] heptane	[201]
21	*Smallanthus sonchifolius *Yacon (Asteraceae)	ferulic acid, chlorogenic acid	[202]
